# Supplementary material for: Ecological properties of soil improved by high–performance ester materials under freeze–thaw cycles conditions
Source: PLoS One. 2025 Jul 1;20(7):e0327417. doi: 10.1371/journal.pone.0327417 (PMC12212570; doi:10.1371/journal.pone.0327417)
Supplement: S1 File — S1 Fig. Relationship between number of FTCs and water–retention capacity. (Note: Data are expressed as mean ± standard deviation (n = 3)). S2 Fig. Relationship between number of FTCs and conductivity. (Note: Data are expressed as mean ± standard deviation (n = 3)). S3 Fig. Relationship between number of FTCs and germination rate. (Note: Data are expressed as mean ± standard deviation (n = 3)). S4 Fig. Relationship between field water–retention capacity and germination rate. (Note: Data are expressed as mean ± standard deviation (n = 3)). S5 Fig. Relationship between conductivity and germination rate. (Note: Data are expressed as mean ± standard deviation (n = 3)). S6 Fig. Relationship between number of FTCs and plant height. S7 Fig. Relationship between field water–retention capacity and plant height. S8 Fig. Relationship between conductivity and plant height. S9 Fig. Sampling location map. S1 Table. Accuracy of model (Relationship between number of FTCs and water–retention capacity). S2 Table. Accuracy of model (Relationship between number of FTCs and conductivity). S3 Table. Accuracy of model (Relationship between number of FTCs and germination rate). S4 Table. Accuracy of model (Relationship between field water–retention capacity and germination rate). S5 Table. Accuracy of model (Relationship between conductivity and germination rate). S6 Table. Accuracy of model (Relationship between number of FTCs and plant height). S7 Table. Accuracy of model (Relationship between field water–retention capacity and plant height). S8 Table. Accuracy of model (S8 Relationship between conductivity and plant height). (ZIP) [file pone.0327417.s001.zip › Supporting information/Supplementary Information.docx]

Supplementary Figure S1 Relationship between number of FTCs and water–retention capacity. (Note: Data are expressed as mean ± standard deviation (n=3))

Supplementary Figure S2 Relationship between number of FTCs and conductivity. (Note: Data are expressed as mean ± standard deviation (n=3))

Supplementary Figure S3 Relationship between number of FTCs and germination rate. (Note: Data are expressed as mean ± standard deviation (n=3))

Supplementary Figure S4 Relationship between field water–retention capacity and germination rate. (Note: Data are expressed as mean ± standard deviation (n=3))

Supplementary Figure S5 Relationship between conductivity and germination rate. (Note: Data are expressed as mean ± standard deviation (n=3))

Supplementary Figure S6 Relationship between number of FTCs and plant height

Supplementary Figure S7 Relationship between field water–retention capacity and plant height

Supplementary Figure S8 Relationship between conductivity and plant height


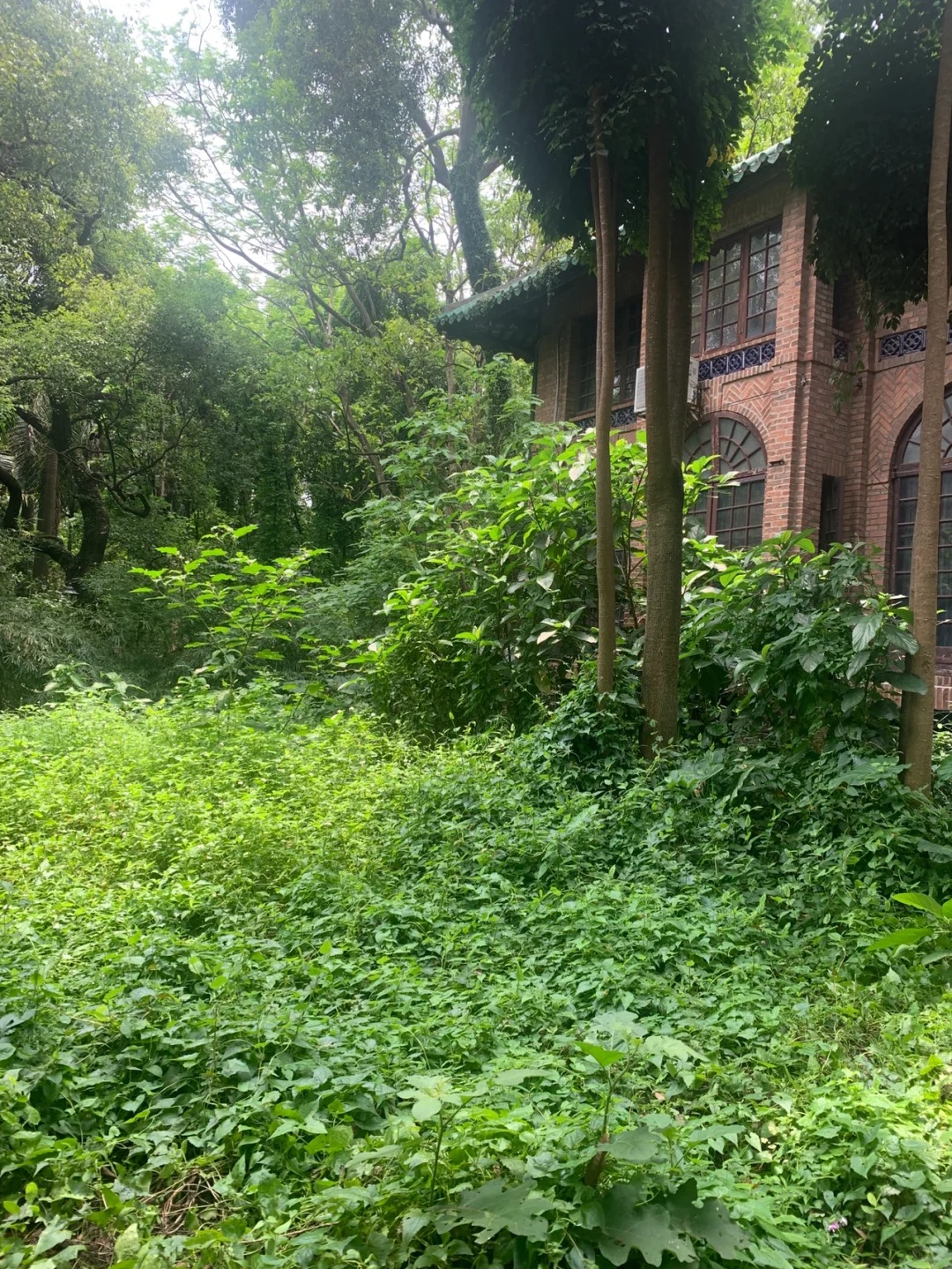


Supplementary Figure S9 Sampling location map

Supplementary Table S1 Accuracy of model (Relationship between number of FTCs and water–retention capacity)

| Group | Standard deviation | Probability >\|t\| | R^2^ |
| --- | --- | --- | --- |
| Water-retaining materials | 4.05921 | 6.43E-18 | 0.93133 |
| Control group | 0.28325 | 6.37E-23 | 0.67767 |
| B-1 | 0.08244 | 4.76E-31 | 0.97775 |
| B-2 | 0.11801 | 2.93E-29 | 0.97583 |
| B-3 | 0.0929 | 4.59E-31 | 0.98926 |
| C-1 | 0.21064 | 4.90E-25 | 0.86982 |
| C-2 | 0.27567 | 8.61E-24 | 0.91953 |
| C-3 | 0.24806 | 6.88E-25 | 0.95077 |
| C-4 | 0.27465 | 1.22E-23 | 0.84205 |
| C-5 | 0.47499 | 1.27E-20 | 0.76586 |
| C-6 | 0.66265 | 9.33E-19 | 0.69858 |
| C-7 | 0.39088 | 1.74E-21 | 0.67892 |
| C-8 | 0.53765 | 7.94E-20 | 0.45633 |
| C-9 | 0.44786 | 2.70E-21 | 0.70194 |

Supplementary Table S2 Accuracy of model (Relationship between number of FTCs and conductivity)

| Group | Standard deviation | Probability >\|t\| | R^2^ |
| --- | --- | --- | --- |
| Adhesive materials | 0.88884 | 0.02563 | 0.25864 |
| Control group | 0.53029 | 7.52E-04 | 0.53677 |
| A-1 | 0.33719 | 4.79E-10 | 0.93777 |
| A-2 | 0.24117 | 1.03E-11 | 0.96398 |
| A-3 | 0.3093 | 2.26E-07 | 0.85086 |
| C-1 | 1.1665 | 0.08562 | 0.89413 |
| C-2 | 1.29257 | 0.017 | 0.96832 |
| C-3 | 1.5912 | 0.09709 | 0.97125 |
| C-4 | 1.53775 | 0.71476 | 0.60890 |
| C-5 | 1.22996 | 0.02844 | 0.87524 |
| C-6 | 1.45244 | 0.00753 | 0.79236 |
| C-7 | 1.11037 | 0.00185 | 0.76444 |
| C-8 | 1.64821 | 0.02336 | 0.67412 |
| C-9 | 1.9297 | 0.21405 | 0.44240 |

Supplementary Table S3 Accuracy of model (Relationship between number of FTCs and germination rate.)

| Group | Standard deviation | Probability >\|t\| | R^2^ |
| --- | --- | --- | --- |
| Control group | 0.62347 | 0.00434 | 0.41261 |
| A-1 | 0.71348 | 0.16544 | 0.70720 |
| A-2 | 0.51341 | 0.07958 | 0.64614 |
| A-3 | 0.50522 | 0.00143 | 0.94454 |
| B-1 | 0.7208 | 0.53728 | 0.41670 |
| B-2 | 0.57616 | 0.02944 | 0.54524 |
| B-3 | 0.92462 | 0.38816 | 0.97013 |
| C-1 | 0.83287 | 0.07975 | 0.62414 |
| C-2 | 0.70479 | 0.00196 | 0.47223 |
| C-3 | 0.76212 | 0.07417 | 0.53661 |
| C-4 | 1.01627 | 0.10443 | 0.84411 |
| C-5 | 0.88677 | 0.04463 | 0.87204 |
| C-6 | 0.79715 | 0.00784 | 0.64563 |
| C-7 | 0.86761 | 0.00136 | 0.97764 |
| C-8 | 0.80672 | 0.00317 | 0.68543 |
| C-9 | 0.98105 | 0.00578 | 0.89773 |

Supplementary Table S4 Accuracy of model (Relationship between field water–retention capacity and germination rate.)

| Group | Standard deviation | Probability >\|t\| | R^2^ |
| --- | --- | --- | --- |
| Control group | 2.63303 | 0.00141 | 0.95294 |
| A-1 | 4.48361 | 0.12207 | 0.61022 |
| A-2 | 2.05323 | 0.08812 | 0.59713 |
| A-3 | 1.7089 | 0.00278 | 0.68244 |
| B-1 | 2.97723 | 0.6054 | 0.50470 |
| B-2 | 1.81484 | 0.06625 | 0.65181 |
| B-3 | 2.33269 | 0.34706 | 0.60035 |
| C-1 | 3.21883 | 0.07055 | 0.87815 |
| C-2 | 1.75113 | 0.00482 | 0.40444 |
| C-3 | 1.62633 | 0.1706 | 0.67550 |
| C-4 | 3.13831 | 0.03716 | 0.89222 |
| C-5 | 2.28653 | 0.28913 | 0.70140 |
| C-6 | 2.06049 | 0.04845 | 0.96701 |
| C-7 | 3.37584 | 0.01647 | 0.99692 |
| C-8 | 3.72869 | 0.15670 | 0.07634 |
| C-9 | 4.62642 | 0.08644 | 0.79613 |

Supplementary Table S5 Accuracy of model (Relationship between conductivity and germination rate.)

| Group | Standard deviation | Probability >\|t\| | R^2^ |
| --- | --- | --- | --- |
| Control group | 0.21526 | 0.23259 | 0.5803 |
| A-1 | 0.18718 | 0.23735 | 0.75033 |
| A-2 | 0.14082 | 0.24877 | 0.90202 |
| A-3 | 0.19499 | 0.02603 | 0.71925 |
| B-1 | 0.17226 | 0.7578 | 0.63910 |
| B-2 | 0.13457 | 0.01323 | 0.93731 |
| B-3 | 0.15166 | 0.29541 | 0.96011 |
| C-1 | 0.17302 | 0.09652 | 0.65912 |
| C-2 | 0.15789 | 0.19086 | 0.55980 |
| C-3 | 0.12272 | 0.20949 | 0.64404 |
| C-4 | 0.19302 | 0.74682 | 0.63190 |
| C-5 | 0.17880 | 0.26266 | 0.70235 |
| C-6 | 0.16347 | 0.12202 | 0.31102 |
| C-7 | 0.16641 | 0.00961 | 0.73334 |
| C-8 | 0.1698 | 0.49593 | 0.52803 |
| C-9 | 0.21495 | 0.06633 | 0.65051 |

Supplementary Table S6 Accuracy of model (Relationship between number of FTCs and plant height)

| Group | Standard deviation | Probability >\|t\| | R^2^ |
| --- | --- | --- | --- |
| Control group | 0.11617 | 0.78186 | 0.65360 |
| A-1 | 0.05877 | 0.16399 | 0.71640 |
| A-2 | 0.04709 | 0.46594 | 0.30090 |
| A-3 | 0.05629 | 0.13748 | 0.89920 |
| B-1 | 0.06126 | 0.93423 | 0.70890 |
| B-2 | 0.07111 | 0.3076 | 0.80100 |
| B-3 | 0.03747 | 0.1876 | 0.57750 |
| C-1 | 0.18106 | 0.15579 | 0.76950 |
| C-2 | 0.07167 | 0.01585 | 0.73031 |
| C-3 | 0.08897 | 0.02807 | 0.50012 |
| C-4 | 0.08654 | 0.00347 | 0.42994 |
| C-5 | 0.0895 | 0.07447 | 0.53261 |
| C-6 | 0.19155 | 0.18454 | 0.59440 |
| C-7 | 0.13238 | 0.07939 | 0.46711 |
| C-8 | 0.08618 | 0.18352 | 0.60020 |
| C-9 | 0.14056 | 0.48472 | 0.33400 |

Supplementary Table S7 Accuracy of model (Relationship between field water–retention capacity and plant height)

| Group | Standard deviation | Probability >\|t\| | R^2^ |
| --- | --- | --- | --- |
| Control group | 0.84808 | 0.25059 | 0.82802 |
| A-1 | 0.36700 | 0.10862 | 0.43611 |
| A-2 | 0.18638 | 0.42169 | 0.21440 |
| A-3 | 0.18678 | 0.21969 | 0.41590 |
| B-1 | 0.25179 | 0.86958 | 0.69290 |
| B-2 | 0.21365 | 0.32937 | 0.42001 |
| B-3 | 0.09385 | 0.14998 | 0.80880 |
| C-1 | 0.70603 | 0.16009 | 0.74130 |
| C-2 | 0.17639 | 0.03510 | 0.84522 |
| C-3 | 0.19086 | 0.06638 | 0.64981 |
| C-4 | 0.30700 | 0.01084 | 0.68533 |
| C-5 | 0.22735 | 0.41042 | 0.90201 |
| C-6 | 0.53946 | 0.49978 | 0.92035 |
| C-7 | 0.47529 | 0.38423 | 0.40130 |
| C-8 | 0.76039 | 0.98457 | 0.71400 |
| C-9 | 0.93215 | 0.211185 | 0.52904 |

Supplementary Table S8 Accuracy of model (S8 Relationship between conductivity and plant height)

| Group | Standard deviation | Probability >\|t\| | R^2^ |
| --- | --- | --- | --- |
| Control group | 0.02494 | 0.91301 | 0.82210 |
| A-1 | 0.01583 | 0.40514 | 0.78601 |
| A-2 | 0.01231 | 0.76145 | 0.64140 |
| A-3 | 0.01836 | 0.21171 | 0.53604 |
| B-1 | 0.01447 | 0.84438 | 0.68380 |
| B-2 | 0.01754 | 0.32628 | 0.32002 |
| B-3 | 0.00622 | 0.18263 | 0.60520 |
| C-1 | 0.01669 | 0.50828 | 0.99103 |
| C-2 | 0.01206 | 0.21057 | 0.54520 |
| C-3 | 0.01272 | 0.08796 | 0.85715 |
| C-4 | 0.01630 | 0.22959 | 0.80442 |
| C-5 | 0.01157 | 0.1046 | 0.80813 |
| C-6 | 0.02052 | 0.34957 | 0.80030 |
| C-7 | 0.01129 | 0.05904 | 0.87204 |
| C-8 | 0.00898 | 0.762333 | 0.74760 |
| C-9 | 0.0159 | 0.88404 | 0.75409 |
