# Supplementary material for: Ecological properties of soil improved by high–performance ester materials under freeze–thaw cycles conditions
Source: PLoS One. 2025 Jul 1;20(7):e0327417. doi: 10.1371/journal.pone.0327417 (PMC12212570; doi:10.1371/journal.pone.0327417)
Supplement: Attachment — Submitted filename: Response to Reviewers.docx [file pone.0327417.s003.docx]

Reviewer #1: This paper investigated the ecological property variations of soil improved by high-performance ester materials under freeze-thaw cycle conditions. The viscosity of high-performance ester adhesive materials, the water absorption capacity of water-retaining materials, the field water holding capacity and soil conductivity of improved soil were experimentally investigated. The plant growth and germination were also explored. Generally, the results are good references for the slope soil ecological restoration in cold regions. The writing-up of this manuscript is generally acceptable, but some clarifications and improvements are still necessary. My particular comments are as follows.

**Response:** Thank you very much for your review of this article and your valuable comments. The author has modified the full text one by one according to your Suggestions. The specific modified content and the reply to the question are shown below.

1. Adding a paragraph to describe how the material works in field may help readers to understand the functions of the material.

**Response 1:** Thank you very much for your review of this article and your valuable comments. We have added a paragraph describing how materials work in the field to help readers understand how they function in “4.2 Mechanisms of freeze-thaw cycles for soil improvement by high-performance ester materials”. We have added a paragraph to the “4.2 Mechanisms of freeze-thaw cycles for soil improvement by high-performance ester materials” section that describes how the materials work in the field to help the reader understand how the materials function.

2. The lines in Fig. 5(a) cannot be distinguished. Legends are necessary.

**Response 2:** Thank you very much for your review of this article and your valuable comments. We have added legends to fig5.

3. Figures 7, 8, 10 and 11 are difficult for readings. Are they possible to change to plane figures?

**Response 1:** Thank you very much for your review of this article and your valuable comments. We have modified Figures 7, 8, 10 and 11.

4. English and presentations should be significantly improved for clear and correct statements or expressions. Clearly present your new findings in a logical way and write it as a research paper for easy readings.

**Response 1:** Thank you very much for your review of this article and your valuable comments. We have checked and revised the full article for grammar and logic.

Reviewer #2: The manuscript explores a timely and important topic regarding the performance of high-performance ester materials used in ecological soil restoration, with a focus on how freeze–thaw (F-T) cycles affect their efficacy. While the study is of potential interest, the current version has several critical flaws in its conceptual framing, methodological rigor, data interpretation, and scientific writing, which significantly weaken its potential contribution to the field. Below is a detailed evaluation.

**Response:** Thank you very much for your review of this article and your valuable comments. The author has modified the full text one by one according to your Suggestions. The specific modified content and the reply to the question are shown below.

1. The manuscript lacks a clearly articulated scientific hypothesis. The rationale for the study is vague and primarily descriptive. The novelty is overstated: effects of F-T cycles on polymer-amended soils have already been widely studied (as partially cited by the authors themselves).

**Response 1:** Thank you very much for your review of this article and your valuable comments. We have revised the manuscript to more clearly articulate the scientific hypothesis and rationale. While previous studies have examined polymer-amended soils properties, our work specifically investigates high-performance ester materials (HPEMs) in freeze-thaw cycles (FTCs), which differ significantly from conventional polymers in both composition and ecological performance. The key novelty of our study lies in: Focusing on HPEMs that have demonstrated excellent soil improvement properties in tropical regions (where freezing is rare), but whose performance in cold regions with FTCs remains unexplored. We have highlighted its innovativeness in the abstract, introduction, and discussion.

2. The "variation law" referred to in the title and text is never rigorously defined or derived in a meaningful scientific way.

**Response 2:** Thank you very much for your review of this article and your valuable comments. To address this concern, we have revised the manuscript to rigorously define and derive the "variation law" based on experimental data and functional relationships.

3. The F-T protocol (–20°C/25°C, 12h/12h, 15 cycles) is not properly justified in the context of actual field conditions. Why 15 cycles? What regional climate does this simulate? These aspects must be linked to realistic environmental scenarios.

**Response 3:** Thank you very much for your review of this article and your valuable comments. The F-T protocol (–20°C/25°C, 12h/12h, 15 cycles) was carefully selected to simulate realistic environmental conditions in seasonal permafrost regions, particularly in northern China, where such temperature fluctuations are common during winter and early spring. The following justifications support our protocol:

Regional Climate Simulation: The temperature range (–20°C to 25°C) reflects typical diurnal variations in cold regions, where sub-freezing nights and thawing days occur frequently. This range is consistent with meteorological data from northern China, where the average winter temperature can drop to –20°C, and daytime thawing reaches up to 25°C in transitional seasons. Number of Cycles (15 Cycles): The 15 cycles were chosen based on: The average annual F-T occurrences in northern China (approximately 10–20 cycles per year). The degradation cycle of high-performance ester materials (HPEMs), which is 2–3 years. Fifteen cycles represent a reasonable accelerated aging test to evaluate the materials' performance over their service life. Duration of Each Phase (12h/12h): The 12-hour freezing and thawing phases were designed to ensure complete freezing and thawing of the soil samples, mimicking natural conditions where temperatures remain below or above freezing for extended periods.

We have also added to this section, and added the citation.

4. The authors mention three categories of experiments, but the distinction between them is confusing and partially redundant.

**Response 1:** Thank you very much for your review of this article and your valuable comments. To address this concern, we have carefully revised the manuscript to better distinguish and logically connect the three experimental categories (I, II, and III). The modifications ensure that each category serves a unique purpose without redundancy, as outlined below:

Category I (Section 2.2.1): Focuses on the material-level properties of HPEMs (viscosity, water absorption) under FTCs. This establishes the baseline degradation behavior of the materials themselves.

Category II (Section 2.2.1): Examines the soil-level effects of individual HPEM components (adhesive or water-retaining materials) on soil properties (water retention, conductivity, pH) under FTCs. This bridges material degradation to soil performance.

Category III (Section 2.2.1): Investigates composite effects by testing combined HPEM formulations (adhesive + water-retaining materials) on soil properties and plant growth under FTCs. This addresses practical application scenarios.

5. The authors state "three parallel groups" but do not provide standard deviations, error bars, or p-values in any figures or tables. This raises concerns about the reproducibility and robustness of the results.

**Response 5:** Thank you very much for your review of this article and your valuable comments. We have added error bars to the data points and extended descriptions of the data averaging and statistical processing.

In response to the deficiencies you mentioned regarding the statistical analysis of the data, we have completely revised the paper as follows:

Error lines and standard deviation added: Error lines (indicating standard deviation) have been added to all graphs, and the sample size (n=3) has been noted in the figure notes. For example, the graph note for Figure 5 was updated to read “Data are presented as mean ± standard deviation (n=3)”.

Statistical significance analysis: One-way analysis of variance (ANOVA) was performed on key indicators (e.g., viscosity, water-holding capacity, conductivity, germination rate, etc.). Significant differences (p<0.05 or p<0.01) were marked with an asterisk (* or **) in the graphs and statistical descriptions were added in the results section.

Methods: A new description of the statistical analysis was added in “Experimental methods”: “Data are presented as the mean ± standard deviation of three replicated experiments. One-way analysis of variance (ANOVA) was used to test the significance of differences between groups.

Since Figure 12-figure supplement 19 is a redrawing and fitting of Figure 5-figure supplement 11, the error bars are not placed in Figure 12-figure supplement 19 to avoid repetition.

6. Figures are poorly formatted, often without adequate labels, units, or legends. Some figures appear to be redundant (e.g., multiple bar graphs for water retention and conductivity with little additional insight).

**Response 6:** Thank you very much for your review of this article and your valuable comments. We have adjusted and modified the figures throughout the article.

7. There is an excessive number of figures (~20+), many of which do not provide substantial new information. Consider combining or removing non-essential plots.

**Response 7:** Thank you very much for your review of this article and your valuable comments. We have adjusted and modified the figures throughout the article.

8. The manuscript is highly descriptive. There is no microscopic, chemical, or structural analysis to elucidate why or how ester materials degrade under F-T cycles (e.g., SEM, FTIR, DSC).

**Response 8:** Thank you very much for your review of this article and your valuable comments. We sincerely appreciate the reviewer’s insightful comment regarding the need for microscopic, chemical, or structural analysis to elucidate the degradation mechanisms of high-performance ester materials (HPEMs) under freeze-thaw cycles (FTCs). While the current study did not include new SEM, FTIR, or DSC analyses, the proposed mechanisms of HPEM degradation are grounded in the extensive prior research conducted by our group, as referenced in the manuscript (e.g., Liao et al., 2022; Zhang et al., 2024; Zhou et al., 2025).

9. Statements about "material aging" and "hydrophilic group loss" are speculative and unsupported by data.

**Response 9:** Thank you very much for your review of this article and your valuable comments. To address this concern, we have revised the manuscript to provide direct experimental evidence supporting these mechanisms. Specifically, we have referenced our prior SEM and FTIR analyses (cited as references (e.g., Liu et al., 2011; Zhou et al., 2019; Liao et al., 2022; Zhang et al., 2024; Zhou et al., 2025) in the manuscript) to substantiate the observed structural and chemical changes in HPEMs under freeze-thaw cycles (FTCs). These analyses confirmed microcrack formation in polyvinyl acetate matrices and progressive scission of ester bonds (C=O at 1730 cm⁻¹) and hydroxyl groups (3400 cm⁻¹), which align with the described degradation pathways.

10. The choice of pigeon pea is reasonable, but the planting methodology is simplistic. Factors such as light exposure, nutrient control, and planting depth are not controlled or even described.

**Response 10:** Thank you very much for your review of this article and your valuable comments. In response to your comment, we have revised the "Planting experiment" section in the "Experimental methods" subsection (Section 2.2.2) to include detailed descriptions of the controlled conditions. Specifically, we now explicitly state the light exposure parameters (e.g., 12-hour light/dark cycles, References are also cited, Hopper et al., 1994; Delhon et al., 1995), nutrient control measures (e.g., standardized nutrient solution application), and planting depth (e.g., 2 cm uniform depth). These additions ensure transparency and reproducibility of our methodology.

11. Germination and height are the only two parameters observed, which is insufficient to assess "ecological restoration effects" in any meaningful sense.

**Response 11:** Thank you very much for your review of this article and your valuable comments. We appreciate the reviewer's insightful comment regarding the assessment of ecological restoration effects. While germination rate and plant height are indeed limited in fully capturing ecological restoration, our study incorporated multiple complementary indicators to provide a more comprehensive evaluation. Specifically, we measured field water-retention capacity, conductivity, and pH of the improved soils, which are critical parameters reflecting soil nutrient storage, microbial activity, and overall soil health (Sections 3.1.2–3.1.4). These metrics collectively demonstrate the ecological benefits of HPEMs, such as enhanced water retention (39.5% after 15 FTCs, Fig. 6) and improved conductivity (up to 73.2% higher than control, Fig. 7), which directly support plant growth and soil stability.

Additionally, the observed trade-offs between material degradation and plant germination (Fig. 10) align with changes in soil properties, reinforcing the ecological relevance of our findings. We acknowledge that broader ecological metrics (e.g., biodiversity, microbial biomass) could further enrich the study, but our focus on physicochemical and immediate plant responses aligns with the paper’s scope of establishing HPEMs' degradation laws under FTCs. Future work will expand on long-term ecological monitoring.

12. The empirical equations provided (e.g., for water retention vs. cycle number) are simplistic linear regressions without statistical metrics (R², RMSE, etc.). They do not represent fundamental "laws" and should not be interpreted as such.

**Response 12:** Thank you very much for your review of this article and your valuable comments. We appreciate the reviewer’s insightful comment regarding the empirical equations in our manuscript. We acknowledge that the term "variation laws" may have been overly strong, and we have revised the text to more accurately describe these relationships as "empirical trends" or "quantitative relationships."

Major Comments:

1. Study Aim, Hypothesis, and Justification

Please clearly formulate the research hypothesis at the end of the Introduction. Currently, the text speaks in general terms (e.g., "the impact of freeze–thaw cycles was examined"), but it is unclear what specific effects are expected and why.

**Response 1:** Thank you very much for your review of this article and your valuable comments. We have added the research hypothesis in the introduction.

Please clarify how your study differs from previous work cited in the Introduction (e.g., lines 48–84). At present, the novelty of the study is not apparent.

**Response 2:** Thank you very much for your review of this article and your valuable comments. Our study addresses a critical gap in the application of high-performance ester materials (HPEMs) for soil improvement under freeze-thaw cycling (FTC) conditions, particularly in cold regions like northern China. While HPEMs have been extensively studied and applied in tropical and temperate environments (e.g., references Huang et al., 2020, 2022; Liao et al., 2022; Zhang et al., 2024; Zhou et al., 2025), their performance under FTCs—a dominant environmental stressor in cold regions—remains poorly understood. Previous research has focused on tropical and temperate applications of HPEMs, where freeze-thaw effects are negligible (e.g., references Kar et al., 2021; Fang et al., 2023). Our study is the first to systematically investigate how FTCs impact the ecological properties of HPEM-amended soils, addressing a pressing need as ecological restoration projects expand into seasonal permafrost areas.

2. Experimental Design and Justification

Please justify the selection of 15 freeze–thaw cycles (Section 2.2.1). Does this correspond to specific climate data? Which geographic region does this simulate? I recommend including a table or figure comparing the lab-based freeze–thaw conditions with real-world environmental data (e.g., average annual cycles in northern China).

**Response 3:** Thank you very much for your review of this article and your valuable comments. The selection of 15 FTCs was based on the following rationale:

Climate Data Alignment: The average annual FTC frequency in seasonal permafrost regions of northern China ranges from 10 to 20 cycles per year, as documented in regional climate studies (e.g., Yin et al., 2013; Liu et al., 2017). Our choice of 15 cycles simulates a typical winter-spring transition in these areas.

Material Degradation Cycle: HPEMs have a degradation cycle of 2–3 years. Given the annual FTC frequency in northern China, 15 cycles represent the cumulative effect over the material’s service life.

To clarify this in the manuscript, we have added: A new table (Table 4) comparing lab-based FTC conditions with real-world data from northern China (see below). A paragraph in Section 2.2.1 (under "Category I") explicitly linking the cycle number to regional climate and material longevity.

Please clarify why pigeon pea was selected as the sole plant species. Are there comparative data supporting its relevance as an ecological indicator?

**Response 4:** Thank you very much for your review of this article and your valuable comments. We sincerely appreciate the reviewer’s insightful comment regarding the selection of pigeon pea (Cajanus cajan) as the plant species in this study. The choice of pigeon pea was based on extensive prior research conducted by our group, which demonstrated its exceptional adaptability and ecological benefits in soil restoration projects, particularly under challenging environmental conditions (e.g., Liao et al., 2022; Zhang et al., 2024; Zhou et al., 2025).

3. Statistical Analysis and Replication

Please apply statistical analysis (e.g., ANOVA followed by Tukey’s HSD test) to test the significance of differences between groups. Include standard deviations or confidence intervals on all graphs and specify sample size (n) in figure captions. As it stands, the graphs may be misleading, as they do not indicate whether the observed changes are statistically significant or simply observational.

**Response 5:** Thank you very much for your review of this article and your valuable comments.

In response to the deficiencies you mentioned regarding the statistical analysis of the data, we have completely revised the paper as follows:

Error lines and standard deviation added: Error lines (indicating standard deviation) have been added to all graphs, and the sample size (n=3) has been noted in the figure notes. For example, the graph note for Figure 5 was updated to read “Data are presented as mean ± standard deviation (n=3)”.

Statistical significance analysis: One-way analysis of variance (ANOVA) was performed on key indicators (e.g., viscosity, water-holding capacity, conductivity, germination rate, etc.). Significant differences (p<0.05 or p<0.01) were marked with an asterisk (* or **) in the graphs and statistical descriptions were added in the results section.

Methods: A new description of the statistical analysis was added in “Experimental methods”: “Data are presented as the mean ± standard deviation of three replicated experiments. One-way analysis of variance (ANOVA) was used to test the significance of differences between groups.

Since Figure 12-figure supplement 19 is a redrawing and fitting of Figure 5-figure supplement 11, the error bars are not placed in Figure 12-figure supplement 19 to avoid repetition.

4. Data Visualization

Please revise all figures: Include units on all axes (e.g., µS/cm, %, cm). Ensure consistent and clear axis labels and legends.

**Response 6:** Thank you very much for your review of this article and your valuable comments. We have adjusted all figures to ensure that the axis labels and legends are consistently clear.

Reduce redundancy—many figures display similar trends and could be combined as sub-panels (e.g., Fig. 6a and 6b → Fig. 6A/B).

**Response 7:** Thank you very much for your review of this article and your valuable comments. We have combined similar graphs.

Add numeric axis markers and concise group descriptions either as legends or embedded labels.

**Response 8:** Thank you very much for your review of this article and your valuable comments. We have added numeric axis markers and concise group descriptions.

5. Conclusions and Interpretation

Please provide more mechanistic explanations in the discussion—why does viscosity decrease? What specific physicochemical processes are responsible for the degradation of ester materials?

**Response 9:** Thank you very much for your review of this article and your valuable comments. The decrease in viscosity of high-performance ester adhesive materials (HPEMs) under freeze-thaw cycles (FTCs) is attributed to the disruption of polymer chains and the breakdown of physicochemical bonds. In addition to the internal degradation mechanisms previously described, external environmental factors significantly contribute to the degradation of HPEMs under field conditions.

(1) Internal factor:

Microcrack Formation: Repeated FTCs induce thermal stress, leading to microcracks in the polyvinyl acetate matrix. This structural damage reduces the material's cohesive strength, directly impacting viscosity.

Hydrogen Bond Scission: The hydrophilic groups (e.g., hydroxyl and ester bonds) in HPEMs are susceptible to hydrolysis under cyclic freezing and thawing. FTIR analysis (referenced in our prior work (Liu et al., 2011; Zhou et al., 2019; Liao et al., 2022; Zhang et al., 2024) confirmed the cleavage of C=O (1730 cm⁻¹) and O-H (3400 cm⁻¹) bonds, which are critical for maintaining viscosity.

Aggregate Disintegration: The long-chain macromolecules encapsulating soil particles fracture, weakening the elastic membrane structure (Fig. 20). This disintegration is corroborated by SEM observations of aggregate breakdown after 5+ FTCs.

These processes collectively explain the exponential decline in water absorption (due to polymer chain scission) and the linear reduction in viscosity (due to cumulative bond disruption).

(2) External factors:

Soil pH and Ionic Effects: The mild acidification of amended soils (pH 6.6–7.4, Fig. 8) accelerates ester bond hydrolysis via proton-catalyzed reactions, particularly in hydrophilic groups (e.g., −COOH). Cation exchange (e.g., Na⁺ in sodium polyacrylate) with soil ions (e.g., Ca²⁺) disrupts polymer networks, reducing water absorption (Section 3.1.1).

Thermal and Hydration Cycling: Extreme temperatures (−20°C to 25°C, Table 4) induce repeated expansion/contraction stresses, exacerbating microcrack propagation (SEM evidence in Liao et al., 2022; Zhang et al., 2024).

Dry-wet cycles (unfrozen water migration, Section 3.1.2) promote polymer swelling-shrinkage, fragmenting long-chain structures.

Mechanical Abrasion: Soil particle movement during FTCs physically shears polymer coatings (Fig. 20), as observed in SEM images of disintegrated aggregates (Liu et al, 2011; Zhou et al., 2019).

Microbial Activity: While HPEMs are designed for 2–3 year biodegradation (Huang et al., 2020, 2022), cold-region microbial communities may slow degradation. Future studies will quantify microbial contributions via TGA-DSC and enzymatic assays.

These extrinsic factors synergize with intrinsic material properties (e.g., bond cleavage) to drive the observed linear/exponential degradation trends.

Consider including—or at least discussing—the potential role of microscopic or structural analysis (e.g., SEM, FTIR, TGA), even if only as a direction for future research.

**Response 10:** Thank you very much for your review of this article and your valuable comments. We sincerely appreciate the reviewer’s insightful suggestion regarding the potential role of microscopic or structural analysis (e.g., SEM, FTIR, TGA) in our study. While our current research primarily focused on macroscopic ecological and mechanical properties of soil improved by high-performance ester materials (HPEMs) under freeze-thaw cycling (FTC) conditions, we fully acknowledge the importance of microscopic or structural characterization to further elucidate the underlying mechanisms.

In future studies, techniques such as scanning electron microscopy (SEM) could be employed to visualize the morphological changes in HPEMs and soil aggregates before and after FTCs, providing direct evidence of microcrack formation and polymer degradation. Fourier-transform infrared spectroscopy (FTIR) would help identify specific chemical bond scissions (e.g., ester or hydroxyl groups) during FTCs, complementing our current viscosity and water-absorption data. Additionally, thermogravimetric analysis (TGA) could quantify the thermal stability and degradation kinetics of HPEMs under cyclic freezing and thawing.

These analyses would deepen our understanding of the material-soil interactions at a molecular level and refine the quantitative relationships proposed in this study. We have added a brief discussion on this perspective in the Mechanisms of FTCs for Soil Improvement by HPEMs section and will prioritize such investigations in our subsequent work.

The conclusions are too general. Please link your key findings directly to your numerical results, rather than summarizing general trends.

**Response 11:** Thank you very much for your review of this article and your valuable comments. We have modified our conclusions to relate the main findings directly to the numerical results.

6. Language and Style

The manuscript requires thorough language editing by a native English speaker. There are numerous grammatical errors and awkward phrasings, such as:

**Response 12:** Thank you very much for your review of this article and your valuable comments. We have checked the full article grammar and modified it.

“wood bean” instead of “pigeon pea” – possible mistranslation.
**Response 13:** Thank you very much for your review of this article and your valuable comments. It was identified as “pigeon pea”.

“the materials lower pH” – unclear whether this refers to increased acidity or alkalinity.

**Response 14:** Thank you very much for your review of this article and your valuable comments. o clarify, the high-performance ester materials (HPEMs) slightly lowered the soil pH toward neutrality (6.6–7.4) compared to the control group, indicating a mild acidifying effect (Fig. 8). This aligns with the materials’ inherent pH range (6–7) and their interaction with soil cations. To prevent misinterpretation, we have revised the statement in the original text.

Please shorten the abstract. It contains unnecessary repetition and overly specific detail that is better placed in the Results section.

**Response 15:** Thank you very much for your review of this article and your valuable comments. We have streamlined the abstract.

7. Citations and Sources

Please update your references with recent literature from 2020–2024 on biodegradable polymers for soil restoration.

**Response 16:** Thank you very much for your review of this article and your valuable comments. We've updated the date of the citation.

In the methods section, where referring to standards (e.g., the tea bag method, ISO guidelines), please include full source citations rather than vague general descriptions.

**Response 17:** Thank you very much for your review of this article and your valuable comments. We've added references.

Minor Comments

Language and style need substantial improvement. The manuscript contains numerous grammatical errors and awkward phrasings.

**Response 1:** Thank you very much for your review of this article and your valuable comments. We have checked and revised the grammar throughout the article.

Many citations are outdated or missing. For example, recent advances in biodegradable polymers for soil stabilization are not referenced.

**Response 2:** Thank you very much for your review of this article and your valuable comments. We have updated the citation date.

Abstract is overly long and should be more concise, with better separation between motivation, methods, key findings, and implications.

**Response 3:** Thank you very much for your review of this article and your valuable comments. We have streamlined the abstract.

Some methods (e.g., tea bag test, viscometer use) are not cited properly or described in enough detail to be replicable.

**Response 4:** Thank you very much for your review of this article and your valuable comments. We have referenced the above methods and added to the steps.
