# Supplementary material for: Ecological properties of soil improved by high–performance ester materials under freeze–thaw cycles conditions
Source: PLoS One. 2025 Jul 1;20(7):e0327417. doi: 10.1371/journal.pone.0327417 (PMC12212570; doi:10.1371/journal.pone.0327417)
Supplement: Attachment — Submitted filename: Response_to_Reviewers_auresp_2.docx [file pone.0327417.s004.docx]

Reviewer #1: The authors have carefully addressed our comments and improved the quality of this manuscript. I have no more comments.

**Response:** Thank you very much for your review of this article and your valuable comments.

Reviewer #2: Dear authors.
Thank you for your comprehensive answers and correcting the article.
Congratulations

**Response:** Thank you very much for your review of this article and your valuable comments.

Reviewer #3: Comments on the Manuscript
**Response:** Thank you very much for your review of this article and your valuable comments. The author has modified the full text one by one according to your Suggestions. The specific modified content and the reply to the question are shown below.

Abstract:

Are the equations in the abstract necessary? As a rule, they should be avoided.

**Response 1:** Thank you very much for your review of this article and your valuable comments. We have removed formulas (slope and exponential equations) from the abstracts and replaced them with qualitative descriptions of trends (e.g., “linear decline,” “exponential decay”) to conform to journal specifications.

Terminology:

Throughout the manuscript, the term “freeze-thaw cyclings” should be replaced with the correct form “freeze–thaw cycles,” including in all figure captions.

**Response 2:** Thank you very much for your review of this article and your valuable comments. We have replaced the terminology in its entirety, including the title, text, figure headings and notes.

Language and Style:
Lines 130–131: Avoid repetition of the phrase “yellow clay.”
Line 134: Missing period at the end of the sentence.
Lines 156–158: The ethical statement should be moved to the end of the article.
Lines 163–165: Avoid repeating the phrase “were collected.” or “stratified sampling”

**Response 3:** Thank you very much for your review of this article and your valuable comments. We have deleted the duplicate expression. Add period. Move the ethical statement to the “Declarations” section. Optimize sentence structure and remove repetitive words.

Tables and Figures:

All tables should be self-explanatory. Below each table, add notes describing the methods used to determine each parameter and including references to the relevant standards.
Instead of providing a range for sand content, report the mean and standard deviation.

Table 3: Contains a formatting error in the third row.

Figure 5: The unit for viscosity should be “Pa·s,” and “−0.06x” should be shown as an exponent.

Figure 6: Figures should be self-contained; include explanations for the A, B, and C labels in the figure notes. This comment applies to other figures as well.

Figure 7: Clarify the meaning of the asterisks.

Figure 8: The axis label is incorrect and should read “Number of freeze-thaw cycles” instead of “cycling.” Move the axis labels outside the plotting area to avoid overlap with the data. Define the symbol “C.”

Figures 10–11: Correct axis labels as above. For plant height, use [cm] instead of [m], and maintain consistent decimal formatting across all values.

Figure 12: Offers little additional insight and largely duplicates information already shown in Figure 6. Similarly, Figures 14–16 are not interpretable and do not provide significant added value. Reduce the total number of figures to the minimum necessary and place the others in the Supplementary Material. Replace them with a figure that shows the relationship between actual and predicted values to assess model accuracy. Also, report the standard errors (in appropriate units) and R² values for each test group.

Figures 17–19: These are repetitive or unclear and do not make a substantial contribution to the manuscript.

**Response 4:** Thank you very much for your review of this article and your valuable comments.

We have added comments to Tables 1-3 describing the parameter determination criteria.Table 1 Sand content changed to “0.45 ± 0.15%” (mean ± SD). Fixed Table 3 merged cell error.

In Figure 5: Units corrected to “Pa-s”; indices changed to superscript format.

In Figure 6-11: Defined groupings in the notes (A: bonding agent; B: water retaining agent; C: composite material); axes were unified to “Number of freeze-thaw cycles”; unit of plant height was changed to “cm”; and supplemental * indicated significance (p<0.05).

Streamlined graphs: Figures 12-19 moved to Supplementary Materials; added Table S1-S8 to demonstrate model accuracy (R^2^ and standard deviation etc.).

Materials and Methods:
Line 182: Provide details on the equipment used for freeze-thaw cycling, including temperature control specifications.

Line 184: Include the manufacturer's information for the equipment used.

Line 208: Since ANOVA was applied, include a separate “Statistical Analysis” subsection describing the method used, the rationale for using a parametric test, and its application. State whether any post hoc tests were conducted.

Include a results table summarizing the ANOVA analysis—it is currently missing.

Lines 508–517: Each equation should be numbered and followed by explanations of all included variables.

Line 298: Rather than simply referencing ISO standards and methods, briefly describe the methodology and link it to the applicable standards.

A brief subsection on data quality control is missing and should be added to the Materials and Methods section.

**Response 5:** Thank you very much for your review of this article and your valuable comments. We have added the device model (ESPEC PSL-3K) and temperature control accuracy (±0.5°C). Added subsection 2.2.4 “Statistical Analysis” describing ANOVA and Tukey test. Added Supplementary Table S1-S8 (ANOVA results). Number and explain variables for equations (1)-(2). Added a brief description of the methods for determining conductivity (ISSS/SSSA), pH (ISO 10390), and water-holding capacity (FAO 56). Add new subsection 2.2.4 “Statistical Analysis and Data Quality Control”.

Discussion:

Sections 4.1.1 to 4.1.4 lack citations. They currently do not fulfill the role of a scientific discussion. While this may be acceptable in the results section, the discussion must include references to support or contrast the findings.

Avoid using the abbreviation “Figs”—write “Figures” in full.

Lines 662–677: Are the stated conclusions supported by previous studies? A comparative discussion with existing literature is needed.

**Response 6:** Thank you very much for your review of this article and your valuable comments. We have added citations for degradation mechanisms. Replace “Figs” with “Figures” throughout the text. Added comparative discussion (e.g. vs. freeze-thaw response of synthetic polymers).

Other Comments:

The References section should be formatted according to the journal’s submission guidelines.

In Line 164, specify the geographical location of the sample collection site and ideally include a map showing the sampling area.

**Response 7:** Thank you very much for your review of this article and your valuable comments. Our references have been adjusted to the journal format, supplemented with coordinates of the sampling point (23°4′16.3″N, 113°23′29.9″E) and a location map (Supplementary Materials).
